# Effect of High Calorie Diet on Intestinal Flora in LPS-Induced Pneumonia Rats

**DOI:** 10.1038/s41598-020-58632-0

**Published:** 2020-02-03

**Authors:** Chen Bai, Tiegang Liu, Jingnan Xu, Xueyan Ma, Ling Huang, Shaoyang Liu, He Yu, Jianxin Chen, Xiaohong Gu

**Affiliations:** Beijing University of Chinese Medicine, ChaoYang District, Beijing, 100029 China

**Keywords:** Respiratory tract diseases, Paediatric research

## Abstract

Intestinal flora plays an important role in inflammatory response to systemic or local organs of its host. High calorie diet has been shown to aggravate the condition of pneumonia and delay recovery, especially in children. However, the underlying mechanisms remain unclear. This study placed SPF rats in a conventional environment, high calorie diet or LPS atomization was performed respectively or combined. Analysis of high-throughput sequencing of intestinal content combined with animal weight, organ index, serum inflammatory factors indicators and bioinformatics found that after pulmonary infection combined with a high-calorie diet, rats showed significant changes such as weight loss and increased lung weight index, and their lung and intestinal tissues showed more obvious inflammatory changes. And its gut flora structure suggests, the abundance of Leuconostocaceae in significantly reduced; abundance of Staphylococcus, Planococcaceae, Staphylococcus, Staphylococcaceae, Bacillales, Gemellales and Aerococcus significant increased. The study showed that high calorie diet and LPS atomization synergistically promoted pneumonia process in rat pups, which is related to changes in structure of intestinal flora. It is worth noting that pneumonia rats fed by convention diet also causing intestinal flora imbalance.

## Introduction

Long-term high calorie diets have a negative regulatory effect on health. Studies have shown that^1^ high calorie diet may aggravate the condition of pneumonia and delay recovery in respiratory infectious diseases. Changes in the rhythm of modern life caused changes in structure of intestinal flora^2^, such as dietary imbalances^3,4^. It is suggested that changes in dietary composition may cause changes in internal ecosystem of intestine^5^. The healing or aggravation of respiratory inflammation is also related to structure of intestinal flora. For example, the hypothesis theory of “pulmonary-gut axis”^6^ indicates that local inflammatory reactive substances of respiratory system and products of intestinal flora metabolism exchanged and stimulated through lymphatic system and blood circulation system. However, it is still unclear whether intake of high calorie diet aggravates inflammatory response of respiratory tract influenced by intestinal flora. This study explored potential influence of high calorie diet on aggravating pneumonia rats in intestinal flora.

## Materials and Methods

### Animals and reagents

Animals: Clean grade SD rats, 40, male, 4 weeks old, weighing 110 g ± 10 g. Animal certificate No.: 11401500016361, provided by Sibeifu (Beijing) Biotechnology Co., Ltd., license No.: SCXK (Jing) 2016-0002. It is kept in animal laboratory of Institution of Traditional Chinese Medicine, Beijing University of Chinese Medicine with free access to food and water, natural light.

All animal experiments in this study were carried out in accordance with the relevant guidelines and regulations and approved by the Animal Ethics Review Committee of Beijing University of Traditional Chinese Medicine, with the approval number (Animal Laboratory Ethics Examination Number): BUCM-4-20170901-3034.

Homemade feed: according to the ratio of casserole, chocolate wafer, beef grain and flour 1:2:2:1, shape is the same as ordinary feed, and Sibeifu (Beijing) Biotechnology Co., Ltd. is responsible for standard production and quality control, see Table 1.

**Table 1:** Homemade feed ingredients and nutrition comparison table.

	Homemade special feed	Rat maintenance feed
Energy, kilojoule/ 100 g	1828.12	340
Moisture	10.10%	≤10%
Crude protein	13.73%	≥18%
Crude fat	16.10%	≥4%
Carbohydrate	58.80%	—
sodium	0.44%	—
Crude fiber	Not detected	≤5%
Crude ash	0.83%	≤8%
calcium	Not detected	1~1.8%
Total phosphorus	Not detected	0.6~1.2%

Reagents: Motilin ELISA Kit (Wuhan Huamei, CSB-E08208r), Gastrin ELISA Kit (Wuhan Huamei, CSB-E12743r), Rat Single Factor Detection Kit (Rat IL-6), Rat Single Factor Detection Kit (Rat TNF-α), GRO alpha/KC/CINC1, IL-12 p40, Mouse/Rat Basic Kit, NF-κB primary antibody (CST, 8242), MyD88 (Abeam, ab2064), MD2 (Abcam, ab24182), β -actin (CST, 4970 S), secondary antibody auxiliary reagent 042-206.

### Animal feeding and material collection

Forty male SD rats, weighing 110 g ± 10 g, were divided into 4 groups according to their weight: normal control group (NC), high calorie diet group (model control 1, MC1), pneumonia group (model control 2, MC2), high calorie diet combined with pneumonia group (model control 3, MC3). Rats are placed in cages and free access to food and water. Rats were adaptively bred for three days before starting the experiment. On the first day of formal experiment, the MC1 group and the MC3 group were given homemade feed, and daily milk solution was administered; the NC group and the MC2 group were given normal feed, and daily pure water was administered. On the 4th day, MC2 group and MC3 group were sprayed with LPS solution (0.5 mg/ml) one time a day for 30 min; NC group and MC1 group were given equal dose of pure water atomization; all groups were given ordinary feed; stop intragastric administration. See Table 2. After atomization of the sixth day was performed, fasting but still free to water. On the seventh morning, the rats were anesthetized by intraperitoneal injection of chloral hydrate. The blood drawing of abdominal aorta was performed, and then centrifuged at 3500 rpm for 10 min, and serum was taken and stored in a refrigerator at −80 °C. The ice-free liver, spleen, kidney and lung of rats were washed up by physiological saline. The surface moisture was dried up by absorbent paper and then weighed and record. The left lung was placed in a 4% formaldehyde fixative, and stored at 4 °C; the right lung was placed in a frozen pipe and frozen by liquid nitrogen, then stored in a refrigerator at −80 °C.

**Table 2:** Animal treatment methods.

Group	Full name and abbreviation	Quantity	Feed	Atomization
normal control group	normal control, NC	10	normal	pure water
high calorie diet group	model control 1, MC1	10	homemade	pure water
pneumonia group	model control 2, MC2	10	normal	LPS
high calorie diet combined with pneumonia group	model control 3, MC3	10	homemade	LPS

### Detection of sign

Every morning, before the model was performed (materials was taken), the rats was weighed as the weight data for that day. The natural perimeter of rat’s waist is its circumference. The temperature of rat’s left forelimb axillary and rectal was measured by an infrared temperature instrument. Take ratio of a certain organ weight to body weight of rats, that is, the organ-body ratio. The formula is:

$$\frac{liver,\,spleen,\,kidney\,and\,lung\,weight\,(g)}{body\,weight\,(g)}\times 100 \% =liver,\,spleen,\,kidney\,and\,lung\,weight\,ratio$$

### Testing of tissue

Intestinal tissue and lung tissue transverse sections were prepared according to the routine procedure for preparing tissue wax block-slice-HE staining. Serum motilin and gastrin were detected by ELISA method; serum inflammatory factors were treated with Aimplex method according to the reagent instructions. Using simple western technique, protein content of the sample was detected firstly, and then Western band was simulated drawn.

### Detection of microecological in intestinal contents

The microbial DNA sequence in the sample was detected firstly; OTU classification and biological classification level were identified according to database. According to grouping result, cluster analysis and group difference analysis are performed, and relevant statistical charts were drawn. Finally, based on the results of inflammatory factors, correlation analysis between flora and inflammation was performed, and pathway analysis of flora function annotation was performed.

### Bioinformatics research

Firstly, in Polysearch2 (http://polysearch.cs.ualberta.ca/), select “Given” as “Text”, “Find All associated” as “Genes/Proteins”, and enter “High Calorie” in Query Keyword, and click “Quick Search”, and save the result; enter “Pneumoniia” in Query Keyword, and click “Quick Search”, and save the result. To combine high calorie with pneumonia genes, click “search” in string (https://string-db.org/), and select “Multiple Proteins” on the left, and enter the consensus gene in the List of Names, and click “search” and “continue”, get protein interaction. The relationship table was imported into Cytoscape (V3.6.0), and “MCODE” plugin was used to analyze protein interaction network. In David (V6.7) (https://david-d.ncifcrf.gov/) database, click “Start Aanlysis” to enter gene sequence of core network from MCODE analysis into the list and select “office gene symbol” and “Gene List”, and click “Submit list”. Select species “homo sapiens”, and click “Functional Annotation Chart”, wait for a moment, and select KEGG_PATHWAY in Pathways to analyze path enrichment.

## Results

### General situation of animals

**Figure 1:**
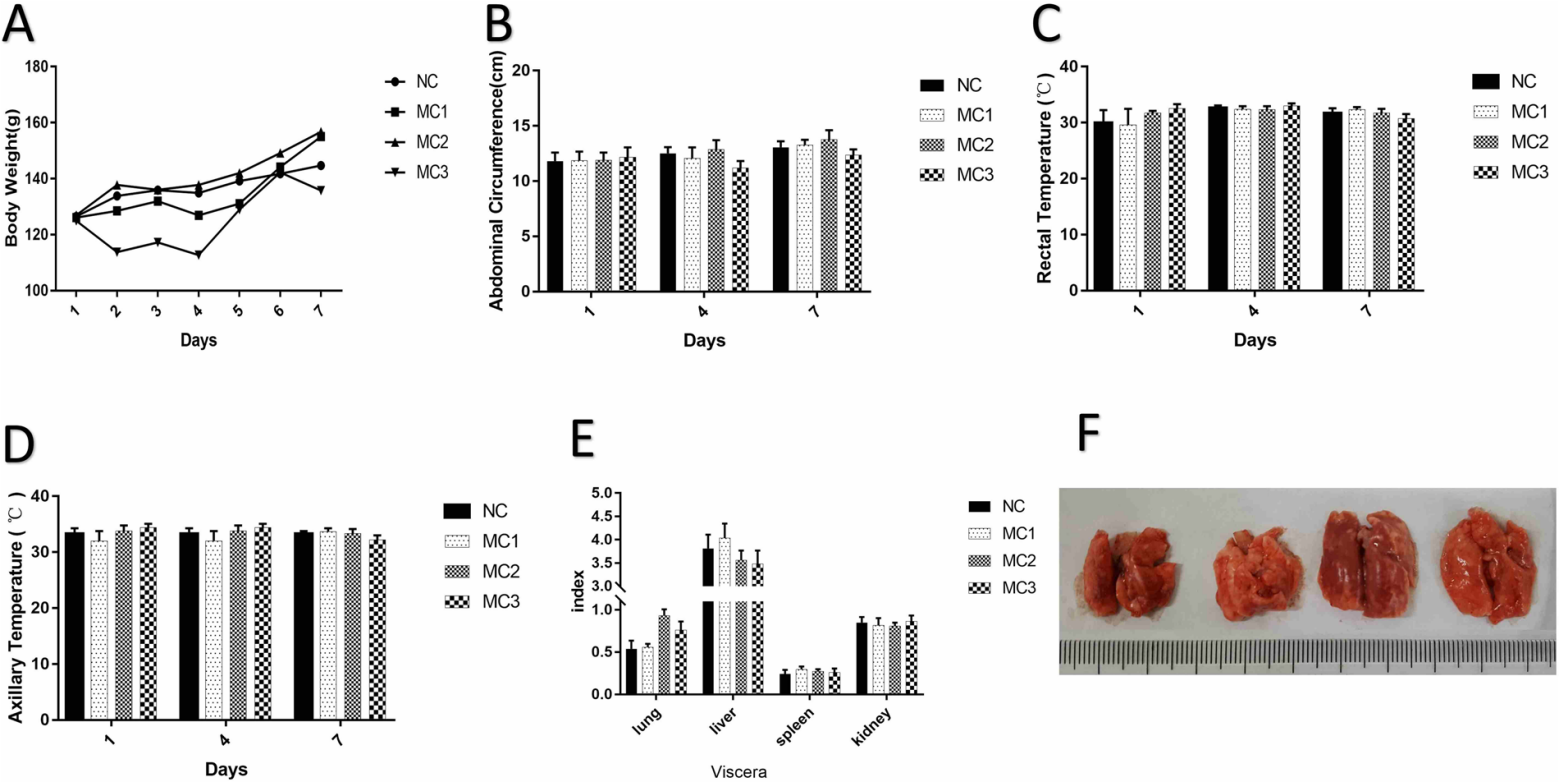
Changes in physical signs of rats with high calorie feed and pneumonia. (**A**) Weight, (**B**) abdominal circumference, (**C**) rectal temperature, (**D**) axillary temperature, (**E**) organ coefficient, (**F**) lung.

During the experiment, the overall weight of four groups of rats showed an increasing trend. Compared with NC group, the body weight of MC3 group decreased after high calorie diet, and the difference was statistically significant. After atomization, the body weight of MC1 and MC2 increased, and of MC3 decreased, the difference was statistically significant (Fig. 1A). Compared with NC group, after high calorie diet, abdominal circumference of each group was not unequal, the difference was statistically significant. After atomization, abdominal circumference of MC2 group increased, and abdominal circumference of MC3 group decreased. The difference was statistically significant (Fig. 1B). Compared with NC group, there was no significant difference in rectal temperature between groups after high calorie diet. The axillary temperature in each group was unequal, and difference was statistically significant. After atomization, the rectal temperature in MC3 group decreased, and the difference was statistically significant (Fig. 1B,C). After the experiment, lung weight coefficient of MC2 and MC3 group increased, and the difference was statistically significant (P < 0.05); liver weight coefficient of MC3 group decreased, and the difference was statistically significant (P < 0.05); spleen weight coefficient of MC1 group increased, and the difference was statistically significant (Fig. 1D,E).

### Serum inflammatory factors and intestinal pattern recognition receptor protein

**Figure 2:**
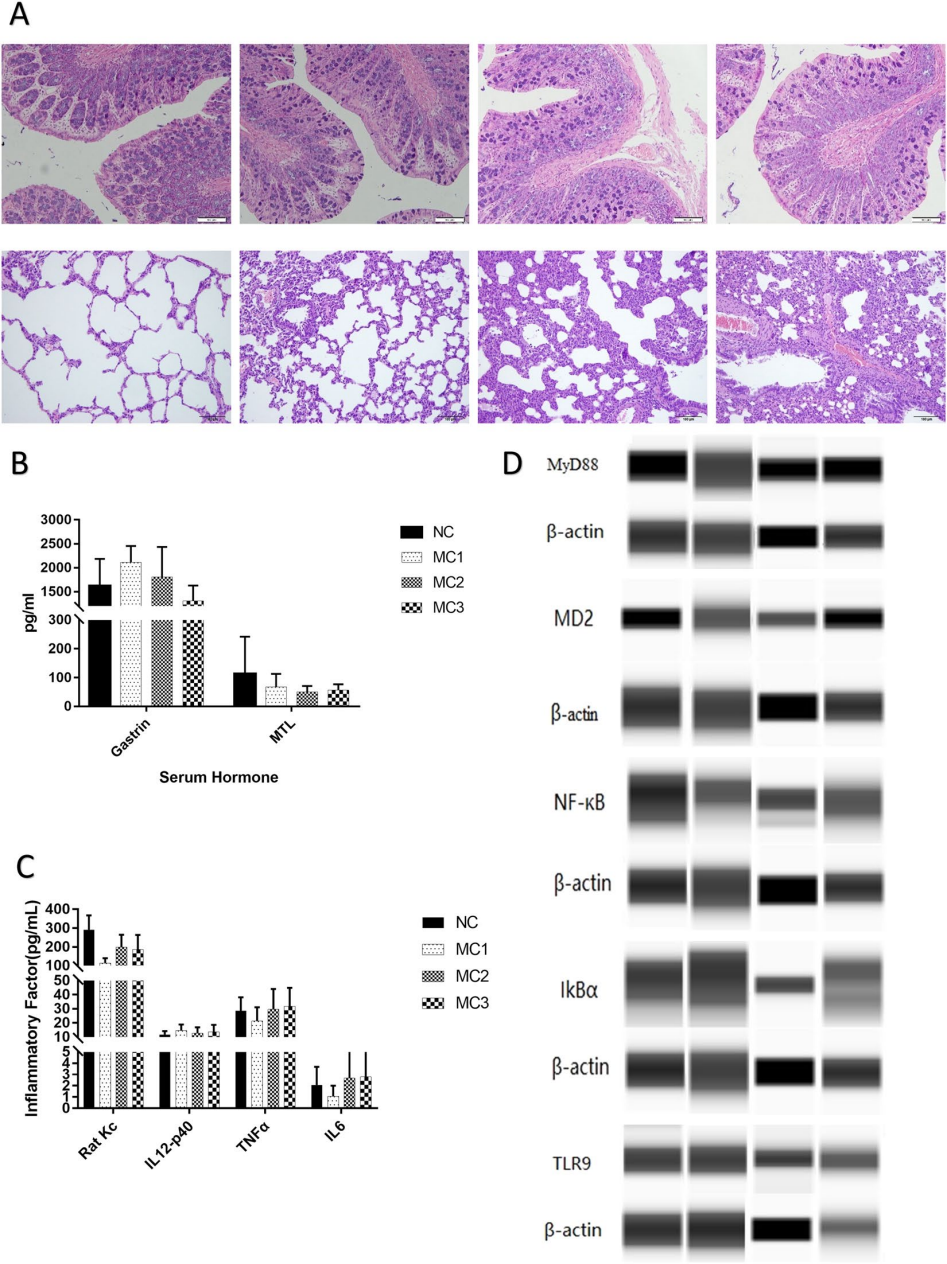
Changes in tissues of rats with high calorie feed and pneumonia. (**A**) HE staining of intestine and lung; (**B**) serum motilin, gastrin; (**C**) serum inflammatory factor; (**D**) recognition receptor of pathway protein in intestinal tissue pattern. From left to right: NC, MC1, MC2, MC3.

After the experiment, colon tissue of NC group and MC2 group was clear by light microscopy; mucosa was smooth and intact; glands were clear and arranged neatly; no inflammatory infiltration, congestion and edema; no cell degeneration and necrosis. In MC1 group and MC3 group, mucosa layer became thinner; number of goblet cells decreased; submucosal lamina propria was locally hyperemic, edematous and fibrotic. The enteraden was multiple, and was straight and long, and arranged tightly. A large number of goblet cells were seen, which were not arranged neatly and tend to enlarge. In NC group, alveolar of lung tissue was intact and the wall was thin. There was no inflamed infiltration of alveolar, and bronchioles were intact and clear. The single-layer columnar or cubic epithelial cells were closely arranged, and epithelial smooth muscle was completely annular. In MC3 group, alveolar structure of lung tissue was unclear, fused or even disappeared; lung interval was significantly widened; vasodilatation and hyperemia; and accompanied by a large number of inflammatory cell infiltration. Compared with MC3 group, lung tissue of MC2 group was milder; alveolar border was clear and the wall was thin; but there was also thickening of lung interval, vascular congestion and a small amount of inflammatory cell infiltration. The single-layer columnar epithelial cells were arranged neatly and tightly (Fig. 2A)

After the experiment, gastrin was unequal in each group, and difference was statistically significant (Fig. 2B); the Kc in each group was unequal, and difference was statistically significant (Fig. 2C). Compared with NC group, MD2 expression was decreased in MC2 group; compared with MC1 group, TLR9 expression was increased in MC3 group, and difference was statistically significant (Fig. 2D).

### Intestinal flora structure

**Figure 3:**
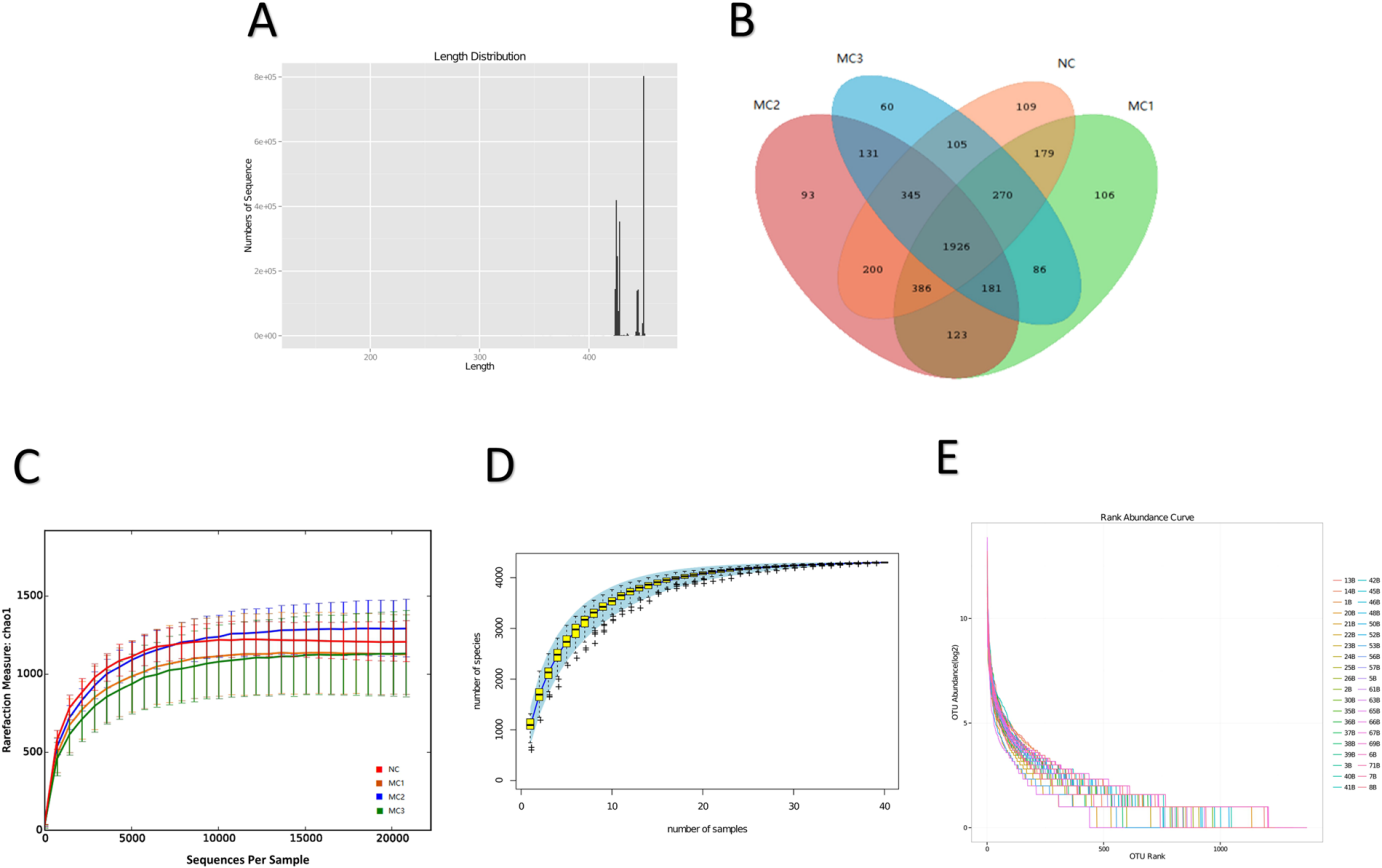
OTU division of intestinal content microecology. (**A**) Sequence length distribution; (**B**) Venn diagram of shared OTU; (**C**) sparse curve of OTU number; (**D**) accumulation curve of specaccum species; (**E**) abundance grade curve; (**F**) microbial number statistics of each classification level.

We performed 40 samples sequencing based on Illumina MiSeq platform, and sequencing length was mainly distributed in 400–500 bp range (Fig. 3A). The results of Venn graph showed that four groups have a total of 1926 OTUs. Among which there are 109 in NC group, 106 in MC1 group, 93 in MC2 group, and 60 in MC3 group (Fig. 3B). The Rarefaction, Species accumulation curves, and abundance registration curves tended to be stable, which indicated that current results are sufficient to reflect diversity of current sample and the average degree is high enough (Fig. 3C,E).

**Figure 4:**
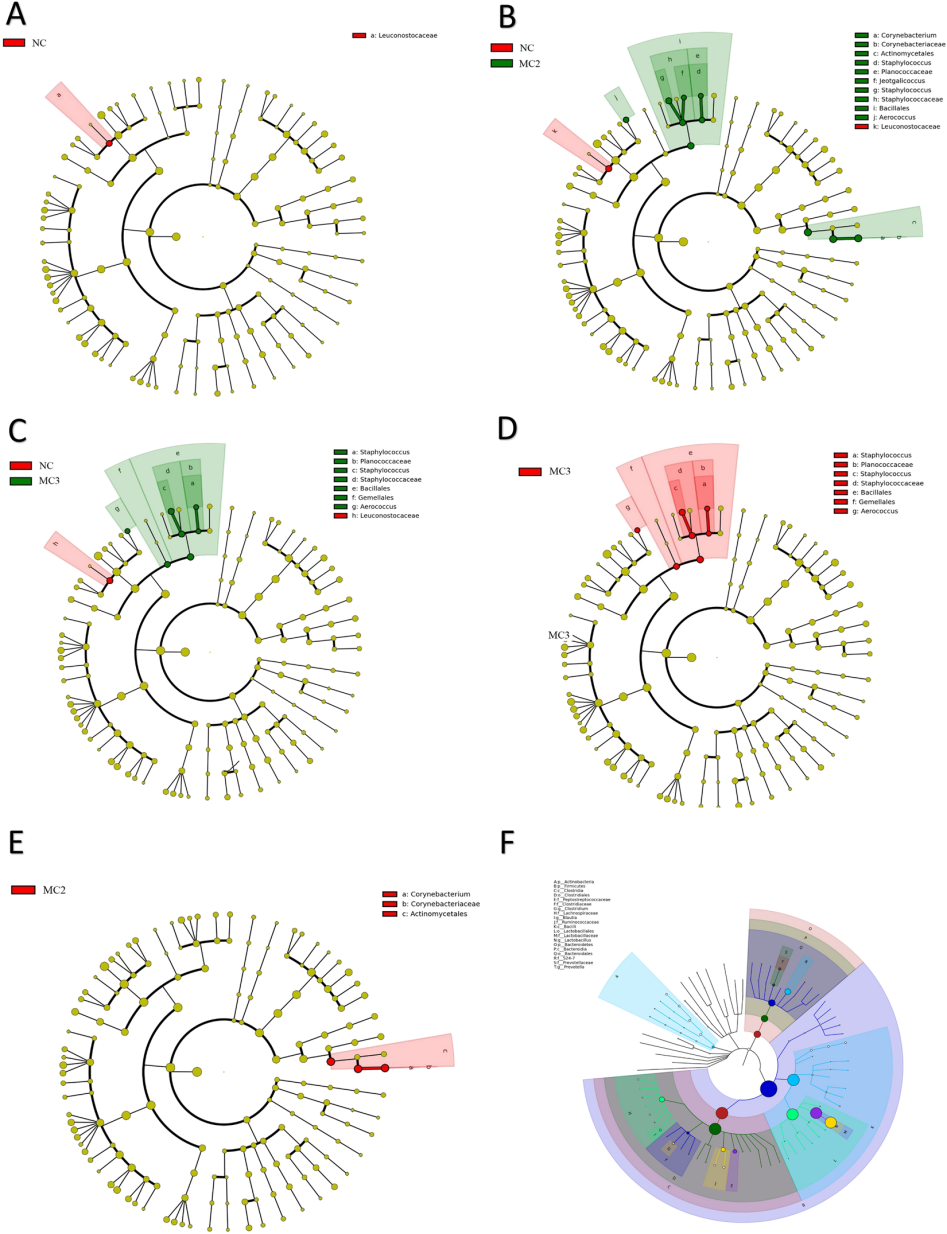
Micro-ecological structure of intestinal contents. (**A**) Group-to-group difference classification unit display map based on classification level tree (NC-MC1); (**B**) group-to-group difference classification unit display map based on classification level tree (NC-MC2); (**C**) group-to-group difference classification unit display map based on classification level tree (NC-MC3); (**D**) group-to-group difference classification unit display map based on classification level tree (MC1-MC3); (**E**) group-to-group difference classification unit display map based on classification level tree (MC2-MC3); (**F**) overall classification level tree diagram based on GrabhlAn sample.

LEfSe analysis was performed by submitting a relative abundance matrix at genus level. Comparison between two groups showed that abundance of Leuconostocaceae in MC1 group was significantly reduced compared with NC group (Fig. 4A); abundance of the Leuconostocaceae in MC2 group was significantly reduced; Corynebacterium, Corynebacteriaceae, Actinomycetales, Staphylococcus, Planococcaceae, Jeotgalicoccus, Staphylococcus, Staphylococcaceae, Bacillales and Aerococcus abundance were significantly increased (Fig. 4B); abundance of Leuconostocaceae in MC3 group significantly reduced; abundance of Staphylococcus, Planococcaceae, Staphylococcus, Staphylococcaceae, Bacillales, Gemellales and Aerococcus significant increased (Fig. 4C); abundance of Staphylococcus, Planococcaceae, Staphylococcus, Staphylococcaceae, Bacillales, Gemelellas, and Aerococcus in MC3 group ware significantly increased compared to MC1 (Fig. 4D); abundance of Corynebacterium, Corynebacteriaceae, and Actinomycetales in MC3 group significantly reduced compared with MC2 group (Fig. 4E). See supplementary data for statistical results. GraPhlAn was used to construct a hierarchical tree for composition of sample population at each taxonomic level. The top 20 abundances were Actinobacteria, Firmicutes, Clostridia, Clostridiales, Peptostreptococcaceae, Clostridiaceae, Clostridium, Lachnospiraceae, Blautia, Ruminococcaceae, Bacilli, Lactobacillales, Lactobacillaceae, Lactobacillus, Bacteroidetes, Bacteroidia, Bacteroidales, S24-7, Prevotellaceae and Prevotella (Fig. 4F).

**Figure 5:**
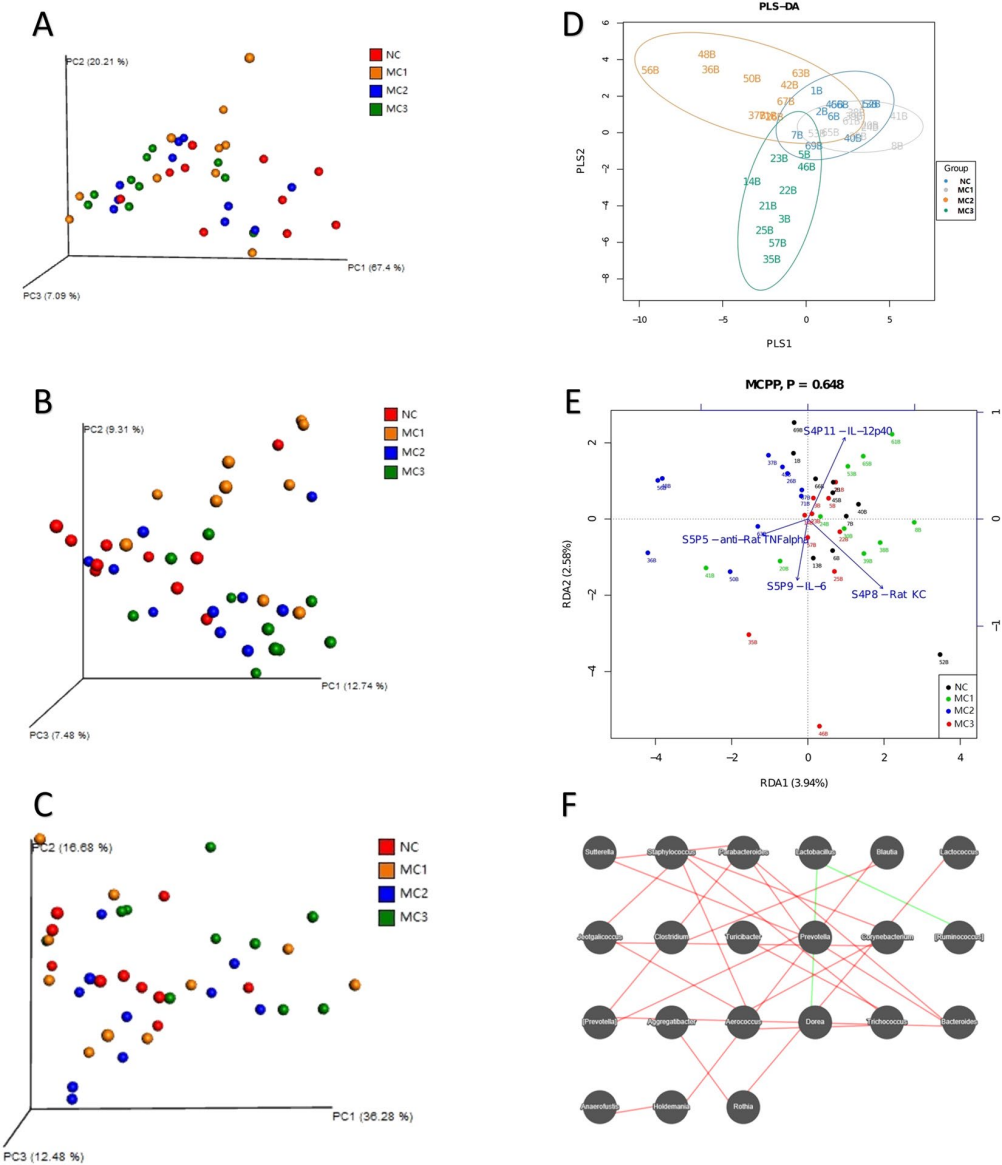
Micro-ecological factors of intestinal contents. (**A**) Three-dimensional sorting map samples of PCA analysis; (**B**) three-dimensional sorting map samples of Unweighted UniFrac PCoA analysis; (**C**) three-dimensional sorting map samples of Weighted UniFrac PCoA analysis; (**D**) PLS-DA discriminant analysis chart; (**E**) RDA constraint sorting chart; (**F**) associated network diagram of dominant genus.

PCA principal component analysis showed that distribution structure of MC2 and NC groups in space was closer; while MC3 and MC1 was a trend distribution in different directions (Fig. 5A). The PCoA principal coordinate analysis showed that difference between samples that leaded MC3 and MC1 to discrete at different angles (Unweighted UniFrac, Fig. 5B); MC3 is more dispersion (Weighted UniFrac, Fig. 5C). Analysis of PLS-DA partial least squares discrimination showed that coincident part of NC group and MC1 group was larger than that of independent part; coincidence part of NC group, MC2 group and MC3 group was smaller than that of independent part; coincidence part of MC1 group and MC3 group was smaller than that of independent part; coincidence part of MC2 group and MC3 group is smaller than that of independent part, which suggesting that classification model works well (Fig. 5D).

Considering the effects of environmental factors on host and flora relationships, this study found that IL-12 and IL8 were significantly more potent than IL-6 and TNF-α. Among which TNF-α-IL-6 and IL-6-IL8 were positively correlation; TNF-α-IL8, TNF-α-IL-12, IL-6-IL-12 and IL8-IL-12 were negatively correlation. MC1 and MC3 were positively correlated with Rat KC and IL-6, while negatively correlated with IL-12 and TNF-α. MC2 was negatively correlated with Rat KC and IL-6, and positively correlated with IL-12 and TNF-α (Fig. 5E). In addition, it is found that a negatively correlation between Lactobacillus-Dorea and Lactobacillus-Ruminococcus in association network of the top 50 abundances and the dominant genus with rho > 0.6 and P value < 0.01 (Fig. 5F).

### Intestinal flora function

**Figure 6:**
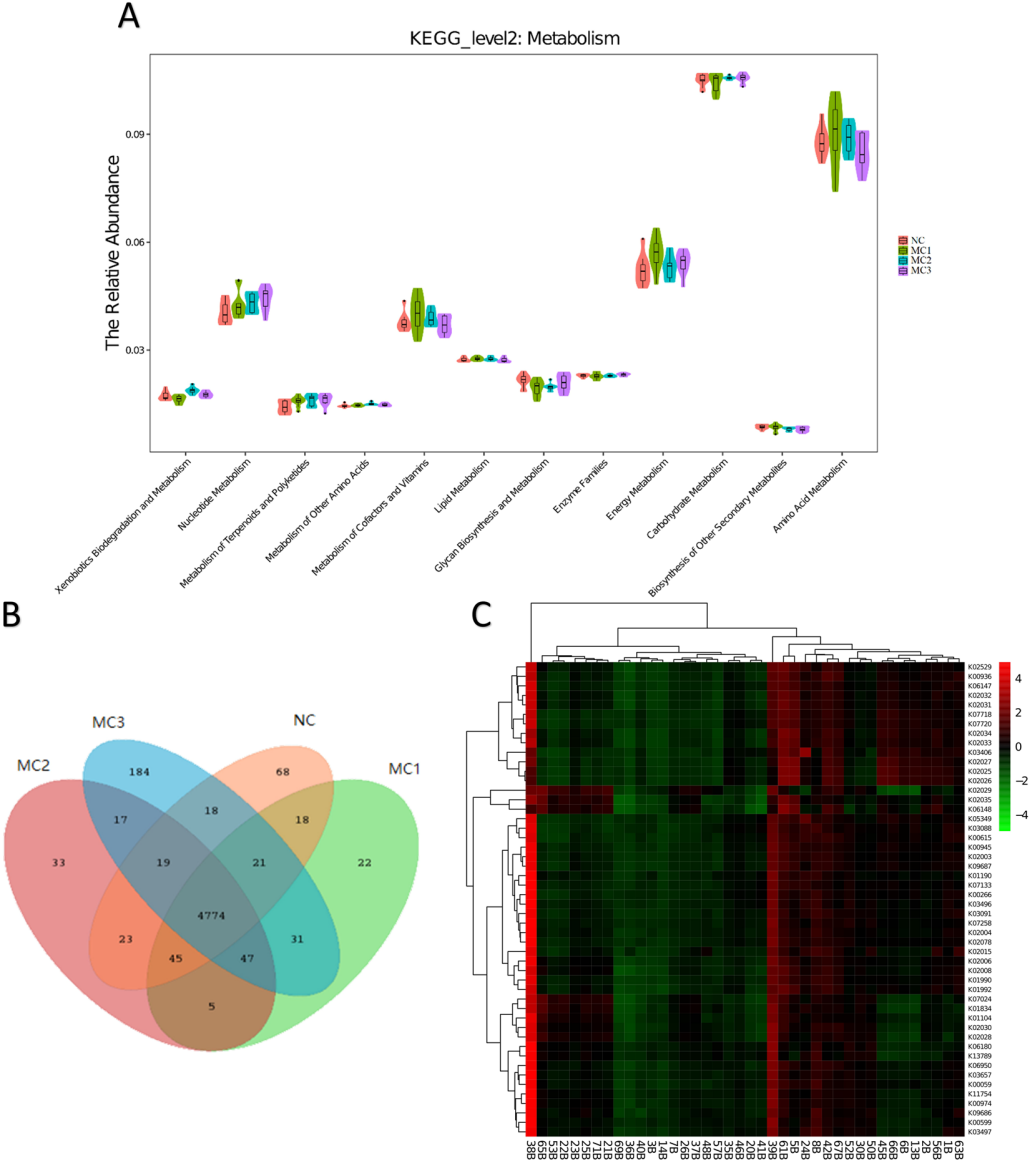
Prediction of metabolic function of intestinal contents. (**A**) Predicted second level distribution diagram of KEGG by KERUSt; (**B**) Venn map of common function group; C: KEGG orthologous gene cluster (KO) abundance heat map combined with cluster analysis.

Based on full-length sequence of 16S rRNA gene of tested microbial genome, and referred to Greengenes 16S rRNA gene full-length sequence database to predict flora metabolic function. KEGG metabolic prediction showed that higher average relative abundance of each function in each sample group was Energy Metabolism, Carbohydrate Metabolism and Amino Acid Metabolism (Fig. 6A). Venn graph results showed that there were 4774 common function in four groups. Among which there were 68 in NC group, 22 in MC1 group, 33 in MC2 group, and 184 in MC3 group (Fig. 6B). The figure results also showed that flora abundance showed different trends after separately or combined the two factors of high calorie diet and LPS atomization (Fig. 6C).

### Construction of biological network

**Figure 7:**
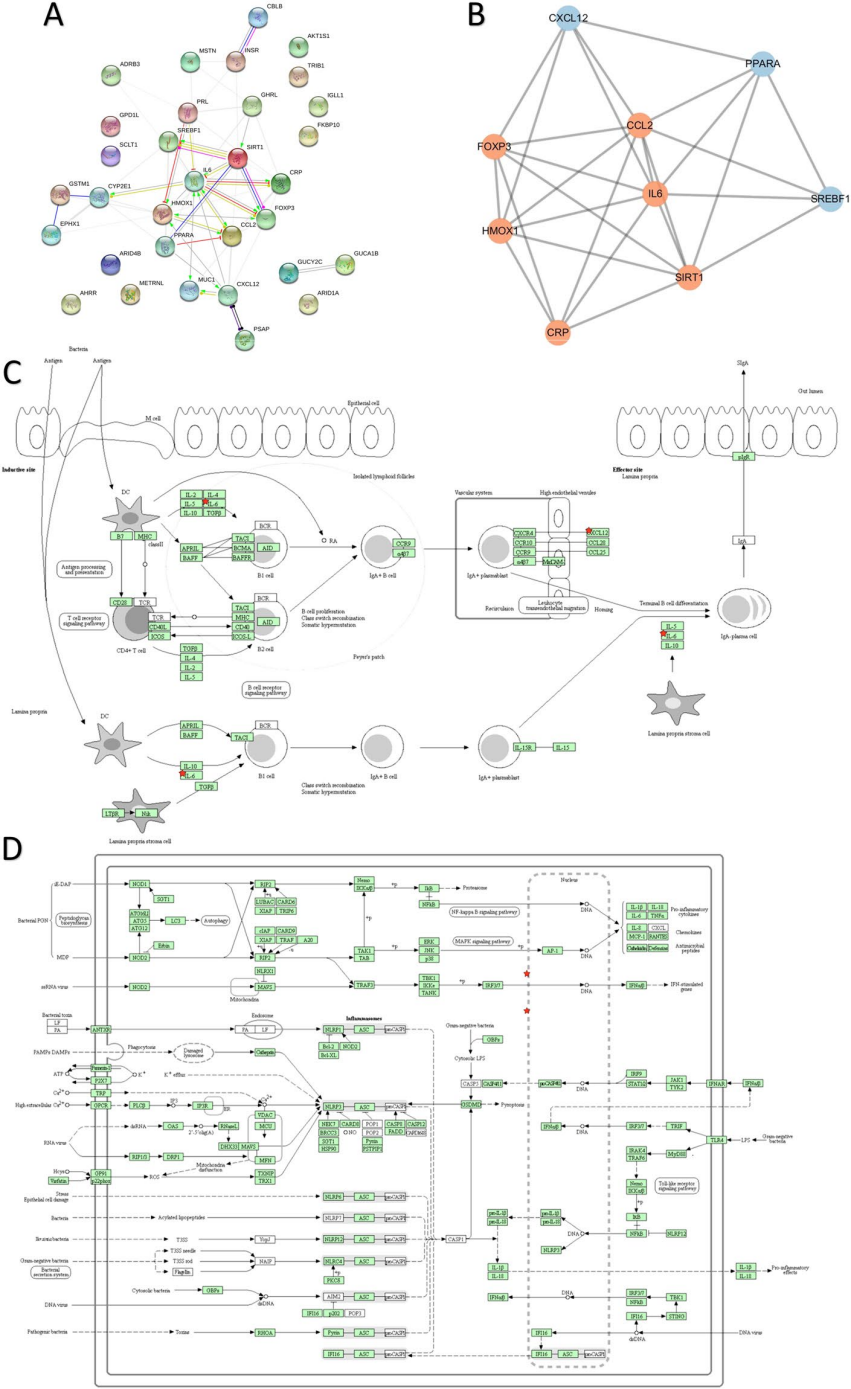
Pathological biological network construction of high calorie diet combined with pneumonia. (**A**) Protein interaction based on string analysis; (**B**) cluster based on mcode analysis (score = 6.75, enrichment is proportional to node shading); (**C**) Enrichment result of Intestinal immune network for IgA production pathway (red star is enrichment protein, degree of protein enrichment is inversely proportional to color shade); (**D**) Enrichment results of NOD-like receptor signaling pathway (red star is enrichment protein, degree of protein enrichment is inversely proportional to color shade).

This study found that eradicate duplicated among 14 genes associated with “High Calorie” and 22 genes associated with “Pneumonia” (see supplementary data) were 35 genes which including 56 sets of protein interactions (Fig. 7A). After clustering, a group of Cluster (Fig. 7B) was obtained. After enrichment, two related pathways were obtained, namely Intestinal immune network for IgA production and NOD-like receptor signaling pathway (Fig. 7C,D).

## Discussion

Pediatric pneumonia is a common disease in children’s respiratory system with a high incidence. It is even one of the important causes of infant and child death in some areas^7–9^. With the changes in social progress and living habits, bad eating habits have become one of the important causes of diseases such as respiratory infections.

**Figure 8:**
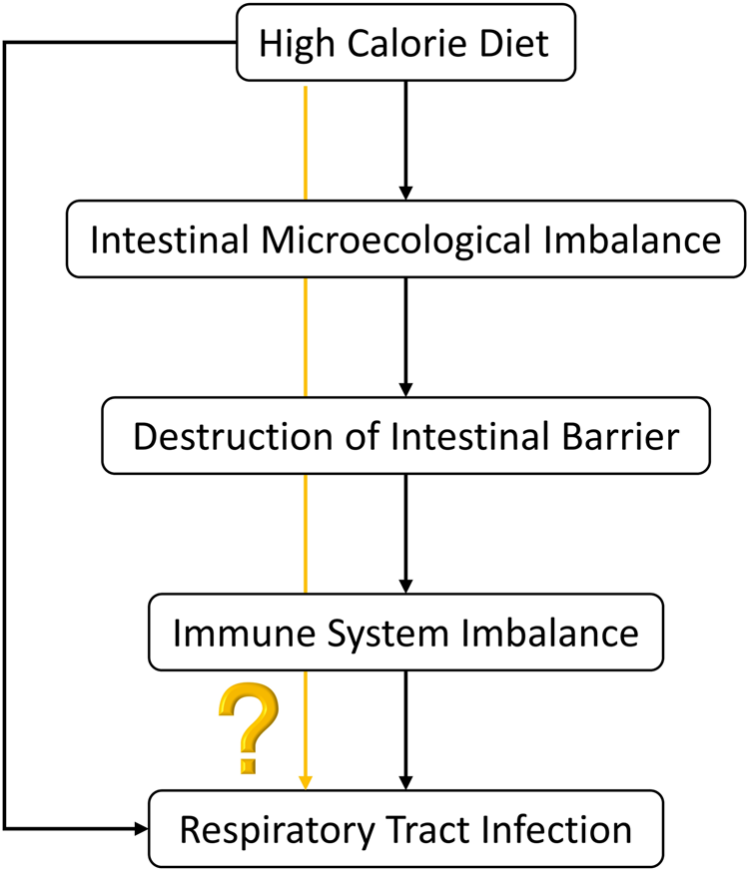
Whether a high-calorie diet affects respiratory tract infections through intestinal flora.

Currently detectable report indicates that incidence of respiratory infections is directly or indirectly related to poor eating habits. In addition to dietary structure, it is also related to gender and underlying diseases (such as metabolic syndrome)^10–12^. The “Five Principles of Healthy Eating” recommended by WHO includes food diversification, salt control, oil control, sugar restriction, and alcohol restriction. While about bad diet, a survey of more than 18,000 American children^13^ pointed out that 60.7% of children drank sugary drinks on a certain day in 2013–2014, although lower than 79.7% in 2003–2004, per capita consumption of sugary drinks for children still maintained an average of 132.5 kcal daily in 2014. According to WHO Guidelines for Adults and Children’s Sugar Intake, free sugar intake reduced to less than 10% of total energy intake. It is also recommended that daily calorie intake should be less than 5% calories per day. The thermal energy supply standard for children aged 4 to 12 years is 1830 to 2470 kcal. Calculated at the lowest value, 5% of total calories are only 91.5 kcal. Experiments have shown that^14^, high calorie diet cause changes in colonic flora and structure and function of animals’ cell. Therefore, correlation between high-calorie diet-intestinal flora-intestinal-lung has become research goal (Fig. 8).

Through animal characterization observation and related indicators, it was found that body weight, abdominal circumference, axillary temperature and rectal temperature of high calorie diet combined with pneumonia showed more obvious changes than high calorie diet groups, suggesting that high calorie diet combined with pneumonia groups was in situation of unbalanced nutrition. The high calorie diet combined with pneumonia showed an increase in organ coefficient of lung and a decrease in organ coefficient of liver, suggesting that LPS-induced lung tissue hypertrophy and liver atrophy occurred in rats’ lung tissue. Analysis of comprehensive nutritional imbalance, it may be LPS infection, and associated with imbalance in rats’ immune function. From pathological results, in addition to high calorie diet significantly aggravated pneumonia, it was also found that rats with simplex high calorie diet had changes in lung tissue inflammation, and rats with simple pneumonia also had mild inflammation in intestinal tissue. Simple pneumonia was caused by LPS, therefore, pathology analysis also suggesting that immune-inflammation interactions between different organs, however, specific pathological mechanism is the commonality between respiratory tract and digestive tract mucosa, or competition between respiratory and digestive system bacteria, further research is still needed.

High-throughput assays showed that two factors of high calorie diets and LPS atomization, respectively or in combination, can cause different degrees of bacterial structural abnormalities in animals, including decreasing in abundance of Leuconostocaceae, or increasing in abundances of a combination with Corynebacterium, Corynebacteriaceae and Actinomycetales. Leuconostocaceae belongs to the group of Lactobacillus and is present in a nutrient-rich environment to ferment glucose by means of mixed fermentation. Lactobacillus can regulate thickness of intestinal mucus layer and expression of mucin-related genes^15–17^ to increase density of intestinal goblet cells and to improve intestinal barrier function and to promote proliferation of intestinal epithelium and to regulate tight junctions between epithelial cells and adhesion link structure^18–20^ so as to play a role of protecting intestinal mucosa. The reduction of probiotics suggested that nutrient content in intestinal environment caused by high calorie diet and/or LPS atomization may be below normal range, which in turn leaded to a decrease in beneficial bacteria represented by lactic acid bacteria in intestine and absence of intestinal protection functions. The majority of bacteria with abundance increasing significantly were not probiotics, which even were pathogenic to host. For example, Corynebacterium can grow and proliferate on mucous membrane of throat and then secrete exotoxin to cause local inflammation and forming a gray-white pseudomembrane; Staphylococcus can invade inhalation and blood infection to cause widespread infection; Bacillus can destroy cell membrane to escape immune recognition, etc.

Intestinal flora affected host’s systemic immunity, such as changing microbial population structure of local environment, which can affect immune function of distal organs. Abt M C *et al*.^21^ pointed out that antiviral innate and adaptive immune responses of dysbacteriosis mice were impaired, and time to clear virus after influenza virus infection was prolonged. Mice macrophage-deficient expression of type I and type II interferons reduced ability to limit viral replication. Schuijt TJ *et al*.^22^ found that C57BL/6 mice after removal of intestinal flora spreading faster, accompanied by severe inflammation and organ failure, and increased mortality after infection with Streptococcus pneumoniae, and weaken engulfment of alveolar macrophages, and reduced response to lipopolysaccharide. It is suggested that intestinal flora may be a protective factor for host to resist pneumococcal disease.

In intestinal tract of pneumonia-infected rats, there is a disorder of intestinal flora, including respiratory pathogens, such as Coryneform bacteria which can cause inflammation through intramural migration; staphylococci can cause cross infection; and Bacillus can reduce immunity, etc. It is suggested that a correlation with micro-ecological system of between intestinal tract and lung. Lung disease causes bacteria migrate to different organs *in vivo*. Its mechanism may be related to lung-based inflammation and immune disorders caused by LPS infection. The lack of lactobacillus also suggested that LPS-induced pneumonia can cause disorder of intestinal flora. It is consistent with clinical application of lactobacillus for pneumonia intervention. However, it needs further study whether the mechanism is competition between bacteria, or interaction between organs through immune-inflammation system. High calorie diet combined with pneumonia group showed a significant reduction in number of Leuconostocaceae; Staphylococcus, Planococcaceae, Staphylococcus, Staphylococcaceae, Bacillales, Gemellales and Aerococcus were significantly increased in high calorie diet combined with pneumonia group. The structure of gastrointestinal heat accumulation combined with pneumonia group is similar to pneumonia group, which showed less pathogenic bacteria. However, axillary bacteria increased in high calorie diet with pneumonia animals compared with normal animals and high calorie diet animals, but not increased in comparison with pneumonia animals, which indicated that bacteria has a correlation the influence of high calorie diet factors and pneumonia induced by LPS. Meanwhile, compared with high calorie diet animals, high calorie diet combined with intestinal disorders of pneumonia animals is mainly caused by virulent cocci, which suggested that it is related to migration and immunity of lungs and intestines. Compared with pneumonia group, reduction of respiratory pathogens such as Coryneform bacteria suggested that high calorie diet caused unsuitable living environment of relevant strains in intestines, which resulted in a significant increase in proportion of pathogenic cocci in intestines and stronger than before. The mechanism may be competition between strains, or may be due to dietary factors that caused changes in intestinal environment, including chemical levels, acid-base balance, etc.

Intestinal flora can affect sensation and motility of intestine directly or indirectly through metabolites, therefore, affect intestinal immune function. TLRs is an important type I transmembrane pattern recognition receptor with same structure, which can recognize structures widely expressed by various pathogens such as lipids, carbohydrates, peptides and nucleic acids. Results of the experiment showed that high calorie diet and pneumonia, simplex or in combination, caused changes in animal serum inflammatory factors and expression of intestinal pattern recognition receptor-related proteins. Animal serum inflammatory factors were elevated; IL-6 can enhance effect of other cytokines; IL-8 can attract and activate neutrophils; IL-12 can act on T cells and NK cells; TNF-α can resist infection and action as an endogenous heat source; RDA redundancy analysis of intestinal contents of four groups animals suggested that in structure of microorganism, high calorie diet combined with pneumonia enhanced effect of cytokines leading to inflammation and inhibits autoimmunity. High calorie diet combined with pneumonia animal pattern recognition receptor protein 9 increased expression, while expression of inflammation-related protein decreased, suggesting that animal showed no serious inflammation level disorder, and intestinal immune-inflammation inhibition. Since TLR9 recognized bacterial CpG-DNA and activated immune-stimulatory properties of B cells and APCs, the response is associated with bacterial flora disorders. Compared with high calorie diet group, Kc and MD2 were elevated in high calorie diet with pneumonia, and IL-12 was decreased, which was opposite to that of normal control group. Compared with pneumonia group, the high calorie diet combined with pneumonia animal showed increase of MD2, NFκB and IkBαMyD88; an opposite trend compared with normal control group, suggested that levels of related inflammatory factors and protein expression were caused by common factors of high calorie diet and pneumonia. Through correlation analysis between sample flora and inflammatory factors, it can be seen that high calorie diet can cause disorder of pneumonia inflammatory factor levels and change the level of inflammatory factor expression. Such as changed positive and negative correlation of serum interleukin, or changed degree of correlation. Annotation analysis of pathway function involved in flora, it was found that flora disorder caused by high calorie diet was related to metabolism of nutrients such as amino acids, carbohydrates, sugar, lipids and vitamins, and also affected enzyme and energy. Therefore, the flora affects receptor protein by affecting intestinal pattern to change level of inflammatory factors and to affect inflammation of lungs, which may affect metabolism of body’s nutrients, energy supply, immune level reduction, and then aggravated pulmonary inflammation.

Life is a complex and dynamic system. It is difficult to control global or core mechanisms by using traditional biomedical research methods to quantitatively or qualitatively study a certain pathological process. Bio-informatics and other biological networks, such as Intestinal immune network for IgA production and NOD-like receptor signaling pathways presented in this study, which may be important pathological mechanisms for high calorie diet combined with pneumonia. However, it still needs to be verified by experiments. In addition, certain complementary traditional medicines, such as Chinese medicine, have unique treatment ideas for related diseases. A prospective cohort study based on TCM theory^23^ indicated that children with gastrointestinal heat may be more susceptible to respiratory infections, where stool dryness and body mass index are positively/negatively associated with recurrent respiratory infections. The relationship between syndrome differentiation system of heat syndrome in Chinese medicine and diet structure, and the impact on respiratory tract infection can be used as an entry point or a breakthrough point for further researches.

In summary, microecological study of high calorie diet pneumonia model is the first report of pneumonia-intestine microecology study based on dietary factors. In view of complexity of dietary structure caused by various regions, races, genders, lifestyles, etc., this study laid the foundation for further study of effects of diet on respiratory tract infections and hotspot study of intestinal flora as a pathogenesis/therapeutic target. Meanwhile, it is of great significance for further research on improving dietary structure and replacing antibiotics for infection.

## Supplementary information

Dataset 1–3.
