# Supplementary material for: Effect of High Calorie Diet on Intestinal Flora in LPS-Induced Pneumonia Rats
Source: Sci Rep. 2020 Feb 3;10:1701. doi: 10.1038/s41598-020-58632-0 (PMC6997398; doi:10.1038/s41598-020-58632-0)
Supplement: Supplementary file 1 — Dataset 1–3. [file 41598_2020_58632_MOESM1_ESM.docx]

**Effect of High Calorie Diet on Intestinal Flora in LPS-Induced Pneumonia Rats**

**Chen Bai^#^, Tiegang Liu^#^, Jingnan Xu, Xueyan Ma, Ling Huang, Shaoyang Liu, He Yu, Jianxin Chen^*^, Xiaohong Gu^*^**

Table 1 List of LEfSe statistical test results (with differences)

| Comparison | | classification list | relative abundance mean in the highest group | group | LDA score | P value |
| --- | --- | --- | --- | --- | --- | --- |
| NC-MC2 | Bacteria.Firmicutes.Bacilli.Bacillales.Planococcaceae | | 3.426965 | MC2 | 3.416172 | 5.38E-05 |
|  | Bacteria.Firmicutes.Bacilli.Bacillales | | 4.06617 | MC2 | 3.824941 | 0.000157 |
|  | Bacteria.Firmicutes.Bacilli.Lactobacillales.Aerococcaceae.Aerococcus | | 3.151072 | MC2 | 3.584472 | 5.38E-05 |
|  | Bacteria.Actinobacteria.Actinobacteria.Actinomycetales.Corynebacteriaceae | | 4.640661 | MC2 | 4.381821 | 9.42E-05 |
|  | Bacteria.Firmicutes.Bacilli.Lactobacillales.Leuconostocaceae | | 3.827191 | NC | 3.511183 | 0.000128 |
|  | Bacteria.Firmicutes.Bacilli.Bacillales.Staphylococcaceae.Jeotgalicoccus | | 3.019773 | MC2 | 3.871276 | 5.38E-05 |
|  | Bacteria.Firmicutes.Bacilli.Bacillales.Staphylococcaceae.Staphylococcus | | 3.860849 | MC2 | 3.704202 | 7.41E-05 |
|  | Bacteria.Actinobacteria.Actinobacteria.Actinomycetales.Corynebacteriaceae.Corynebacterium | | 4.640661 | MC2 | 4.381923 | 9.42E-05 |
|  | Bacteria.Firmicutes.Bacilli.Bacillales.Planococcaceae.Staphylococcus | | 2.623615 | MC2 | 3.635004 | 5.38E-05 |
|  | Bacteria.Firmicutes.Bacilli.Bacillales.Staphylococcaceae | | 3.923001 | MC2 | 3.748611 | 7.41E-05 |
|  | Bacteria.Actinobacteria.Actinobacteria.Actinomycetales | | 4.644815 | MC2 | 4.380321 | 0.000157 |
| NC-MC1 | Bacteria.Firmicutes.Bacilli.Lactobacillales.Leuconostocaceae | | 3.827191 | NC | 3.507752 | 7.41E-05 |
| NC-MC3 | Bacteria.Firmicutes.Bacilli.Bacillales.Planococcaceae | | 2.841122 | MC3 | 3.005675 | 5.38E-05 |
|  | Bacteria.Firmicutes.Bacilli.Bacillales | | 4.047512 | MC3 | 3.737335 | 0.000157 |
|  | Bacteria.Firmicutes.Bacilli.Lactobacillales.Aerococcaceae.Aerococcus | | 2.658772 | MC3 | 3.085768 | 5.38E-05 |
|  | Bacteria.Firmicutes.Bacilli.Lactobacillales.Leuconostocaceae | | 3.827191 | NC | 3.499788 | 0.000148 |
|  | Bacteria.Firmicutes.Bacilli.Bacillales.Staphylococcaceae.Staphylococcus | | 3.992167 | MC3 | 3.718273 | 7.41E-05 |
|  | Bacteria.Firmicutes.Bacilli.Bacillales.Planococcaceae.Staphylococcus | | 2.632201 | MC3 | 2.9402 | 5.38E-05 |
|  | Bacteria.Firmicutes.Bacilli.Gemellales | | 3.588437 | MC3 | 3.391015 | 7.41E-05 |
|  | Bacteria.Firmicutes.Bacilli.Bacillales.Staphylococcaceae | | 3.999911 | MC3 | 3.722612 | 7.41E-05 |
| MC1-MC3 | Bacteria.Firmicutes.Bacilli.Bacillales.Planococcaceae | | 2.841122 | MC3 | 2.887933 | 5.38E-05 |
|  | Bacteria.Firmicutes.Bacilli.Bacillales | | 4.047512 | MC3 | 3.717342 | 0.000157 |
|  | Bacteria.Firmicutes.Bacilli.Lactobacillales.Aerococcaceae.Aerococcus | | 2.658772 | MC3 | 2.888437 | 5.38E-05 |
|  | Bacteria.Firmicutes.Bacilli.Bacillales.Staphylococcaceae.Staphylococcus | | 3.992167 | MC3 | 3.712127 | 7.41E-05 |
|  | Bacteria.Firmicutes.Bacilli.Bacillales.Planococcaceae.Staphylococcus | | 2.632201 | MC3 | 2.769954 | 5.38E-05 |
|  | Bacteria.Firmicutes.Bacilli.Gemellales | | 3.588437 | MC3 | 3.353561 | 5.38E-05 |
|  | Bacteria.Firmicutes.Bacilli.Bacillales.Staphylococcaceae | | 3.999911 | MC3 | 3.719036 | 7.41E-05 |
| MC2-MC3 | Bacteria.Actinobacteria.Actinobacteria.Actinomycetales.Corynebacteriaceae | | 4.640661 | MC3 | 4.344709 | 0.000156 |
|  | Bacteria.Actinobacteria.Actinobacteria.Actinomycetales.Corynebacteriaceae.Corynebacterium | | 4.640661 | MC3 | 4.344709 | 0.000156 |
|  | Bacteria.Actinobacteria.Actinobacteria.Actinomycetales | | 4.644815 | MC3 | 4.339967 | 0.000157 |

Table 2 search results of pneumonia genes/targets

| **ZScore** | **RScore** | **Entity ID** | **Name** | **Synonyms** | **Hits** |
| --- | --- | --- | --- | --- | --- |
| 17.29 | 410 | PS08653 | CAP1A | CAP1A; HCAP-1; HCAP1; HCE-1; HCE1; Human capping enzyme 1; RNA Guanylyl Transferase and 5-prime-Phosphatase; RNA guanylyltransferase and 5'-phosphatase; RNA guanylyltransferase and 5' phosphatase; RNG... (Read More) | 36 [0, 2, 7, 27] |
| 7.41 | 200 | PS23109 | HMOX-1 | HMOX-1; HMOX1; HMOX1 protein; HO-1; Heme oxygenase (Decycling) 1; Heme oxygenase (Decyclizing) 1; Heme oxygenase 1; bK286B10; HMOX1 proteins; Heme oxygenase (Decycling) 1s; Heme oxygenase (Decyclizing... (Read More) | 1 [1, 0, 0, 0] |
| 5.06 | 150 | PS18357 | C reactive protein | C reactive protein; PTX-1; PTX1; C-reactive protein precursor; C-reactive protein precursors; C reactive protein, pentraxin related; C-reactive protein, pentraxin-related; pentraxin 1... (Read More) | 18 [0, 0, 3, 15] |
| 5.06 | 150 | PS18781 | AHRR | AHRR; Arylhydrocarbon hydroxylase; CP11; CYP-1; CYP1; CYP1A1; CYPIA-1; CYPIA1; Cytochrome P450 1A1; Cytochrome P450 1A1 variant; Flavoprotein linked monooxygenase; P1-450; P450 form 6; P450-C; P450-P1... (Read More) | 3 [0, 0, 3, 0] |
| 4.12 | 130 | PS00028 | 14.1 | 14.1; CD179B; CD179b antigen; IGL-1; IGL-5; IGL1; IGL5; IGLJ14.1; IGLL; IGLL-1; IGLL1; IGVPB; Ig lambda-5; Immunoglobulin lambda like polypeptide 1; Immunoglobulin lambda-like polypeptide 1 precursor;... (Read More) | 3 [1, 0, 1, 1] |
| 3.18 | 110 | PS22462 | GST class-mu 1 | GST class-mu 1; Glutathione S-alkyltransferase; GST1; GSTM-1; GSTM1; GSTM1 protein; GSTM1-1; GSTM1a-1a; GSTM1b-1b; GTH-4; GTH4; GTM-1; GTM1; Glutathione S-aralkyltransferase; Glutathione S-transferase... (Read More) | 3 [0, 0, 2, 1] |
| 3.18 | 110 | PS23800 | ARID domain-containing protein 1A | ARID domain-containing protein 1A; P270; ARID1A; AT rich interactive domain 1A; AT rich interactive domain 1A (SWI- like); AT rich interactive domain 1A (SWI-like); AT rich interactive domain 1A isofo... (Read More) | 6 [0, 0, 4, 2] |
| 2.94 | 105 | PS17186 | GIG2 | GIG2; Antigen defined by monoclonal antibody 602-29; BTCC-1; BTCC1; CD9 antigen; CD9 antigen (p24); CD9 molecule; DRAP-27; Leukocyte antigen MIC3; MIC3; MRP-1; Motility related protein; TSPAN29; Tetra... (Read More) | 2 [1, 0, 0, 1] |
| 2.71 | 100 | PS22475 | GST class theta | GST class theta; GST class-theta-1; GSTT-1; GSTT1; GSTT1 protein; Glutathione S-transferase theta 1; Glutathione transferase T1 1; glutathione-S-transferase T1; GST class-theta-1s; GSTT1 proteins; Glu... (Read More) | 2 [0, 0, 2, 0] |
| 2.71 | 100 | PS20237 | EPHX | EPHX; EPHX-1; EPHX1; EPHX1 protein; EPOX; Epoxide hydratase; Epoxide hydrolase; Epoxide hydrolase 1; Microsomal epoxide hydrolase; EPHX1 proteins; Epoxide hydratases; Epoxide hydrolases; Epoxide hydro... (Read More) | 2 [0, 0, 2, 0] |
| 2.71 | 100 | PS18803 | CPE-1 | CPE-1; Flavoprotein linked monooxygenase; Xenobiotic monooxygenase; Microsomal monooxygenase; CPE1; CYP2E; CYP2E1; CYP2E1 protein; CYPIIE-1; CYPIIE1; Cytochrome P450 2E1; Cytochrome P450 family 2 subf... (Read More) | 2 [0, 0, 2, 0] |
| 2.47 | 95 | PS18480 | 40 kDa proline-rich AKT substrate | 40 kDa proline-rich AKT substrate; AKT1 substrate 1; AKT1 substrate 1 (proline-rich); AKT1S1; PRAS40; Proline-rich AKT1 substrate 1; AKT1 substrate 1s; AKT1 substrate 1 (proline-rich)s; Lobes; Proline... (Read More) | 15 [0, 0, 1, 14] |
| 2 | 85 | PS18627 | CXCL12 | CXCL12; Chemokine; Chemokine ligand 12; PBSF; Pre B cell growth stimulating factor; SCYB12; SDF 1 alpha; SDF 1 beta; SDF 1a; SDF 1b; SDF-1; SDF1; SDF1A; SDF1B; Stromal cell derived factor 1; Stromal c... (Read More) | 4 [0, 0, 2, 2] |
| 1.77 | 80 | PS07560 | A1 activator | A1 activator; PSAP; Protein C; CSAct; Cerebroside sulfatase activator; Cerebroside sulfate activator; Co-beta-glucosidase; Component C; Dispersin; GLBA; Glucosylceramidase activator; Proactivator poly... (Read More) | 3 [0, 0, 2, 1] |

Table 3 Search results of high calorie genes/targets

| **ZScore** | **RScore** | **Entity ID** | **Name** | **Synonyms** | **Hits** |
| --- | --- | --- | --- | --- | --- |
| 13.86 | 55 | PS10134 | NAD-dependent deacetylase sirtuin-1 | NAD-dependent deacetylase sirtuin-1; SIR2-like protein 1; SIR2L1; SIRT-1; SIRT1; Sir2 like 1; Sir2alpha; Sirtuin 1; Sirtuin type 1; hSIR2; hSIRT1; sirtuin (silent mating type information regulation 2 ... (Read More) | 11 [0, 0, 0, 11] |
| 10.81 | 45 | PS07106 | NR1C1 | NR1C1; PPAR; PPAR alpha; PPARA; PPARalpha; Peroxisome proliferator activated receptor; Peroxisome proliferator-activated receptor alpha; hPPAR; PPAR alphas; PPARalphas; Peroxisome proliferator activat... (Read More) | 5 [0, 0, 1, 4] |
| 9.29 | 40 | PS07437 | Prolactin | Prolactin; Prolactin precursor; Prolactins; Prolactin precursors... (Read More) | 4 [0, 0, 1, 3] |
| 9.29 | 40 | PS22530 | GUANYLIN | GUANYLIN; GUCA-2; GUCA2; GUCA2A; Gap-I; Guanylate cyclase activating protein1; Guanylate cyclase activator 2A; Guanylate cyclase-activating protein 1; Guanylin precursor; STARA; guanylate cyclase acti... (Read More) | 4 [0, 0, 1, 3] |
| 7.76 | 35 | PS21867 | Appetite-regulating hormone | Appetite-regulating hormone; Appetite-regulating hormone precursor; GHRL; Ghrelin; Growth hormone releasing peptide; Growth hormone secretagogue; M46 protein; MTLRP; Motilin related peptide; UNQ524/PR... (Read More) | 3 [0, 0, 1, 2] |
| 4.71 | 25 | PS00410 | HIRS-1 | HIRS-1; HIRS1; IRS 1; IRS1; Insulin receptor substrate 1; Insulin receptor substrate 1s... (Read More) | 1 [0, 0, 1, 0] |
| 4.71 | 25 | PS22537 | GUC2C | GUC2C; GUCY2C; Guanylate cyclase 2C; Guanylyl cyclase 2C; Heat stable enterotoxin receptor; Heat-stable enterotoxin receptor precursor (GC-C) (Intestinal guanylate cyclase); Intestinal guanylate cycla... (Read More) | 5 [0, 0, 0, 5] |
| 4.71 | 25 | PS21797 | GDF-8 | GDF-8; GDF8; Growth differentiation factor 8; Growth/differentiation factor 8 precursor; MSTN; Myostatin; Growth differentiation factor 8s; Growth/differentiation factor 8 precursors; Myostatins... (Read More) | 1 [0, 0, 1, 0] |
| 4.71 | 25 | PS00335 | CD220 | CD220; CD220 antigen; HHF-5; HHF5; INSR; INSR protein; Insulin receptor; Insulin receptor alpha subunit; Insulin receptor beta subunit; Insulin receptor precursor; Insulin receptor variant; Insulin-li... (Read More) | 1 [0, 0, 1, 0] |
| 4.71 | 25 | PS16875 | CBLB | CBLB; Cas-Br-M (murine) ecotropic retroviral transforming sequence b; Cas-Br-M (murine) ectropic retroviral transforming sequence b; Casitas B-lineage lymphoma proto-oncogene b; CblB; E3 ubiquitin-pro... (Read More) | 1 [0, 0, 1, 0] |
| 4.71 | 25 | PS23878 | ARID4B | ARID4B; ARID4B protein; AT rich interactive domain 4B; AT rich interactive domain 4B (RBP1- like); AT rich interactive domain 4B (RBP1-like); AT rich interactive domain 4B RBP1 like; BCAA; BRCAA-1; BR... (Read More) | 1 [0, 0, 1, 0] |
| 4.71 | 25 | PS00184 | 26 kd protein | 26 kd protein; B cell differentiation factor; B cell stimulatory factor 2; BSF-2; BSF2; CTL differentiation factor; Hepatocyte stimulatory factor; Hybridoma growth factor; IFNB-2; IFNB2; IL-6; Interfe... (Read More) | 5 [0, 0, 0, 5] |
| 3.19 | 20 | PS02410 | METRNL | METRNL; METRNL protein; Meteorin glial cell differentiation regulator like; METRNL proteins; Meteorin glial cell differentiation regulator likes; Hypothetical protein LOC284207; meteorin, glial cell d... (Read More) | 4 [0, 0, 0, 4] |
| 3.19 | 20 | PS22174 | GPD1L | GPD1L; glycerol-3-phosphate dehydrogenase 1-like; glycerol-3-phosphate dehydrogenase 1-likes; KIAA0089... (Read More) | 4 [0, 0, 0, 4] |
| 3.19 | 20 | PS17067 | CCL-2 | CCL-2; MCAF; CCL2; GDCF-2; GDCF-2 HC11; GDCF2; HC11; HSMCR30; MCP-1; MCP1; Monocyte chemoattractant protein 1; Monocyte chemotactic and activating factor; Monocyte chemotactic protein 1; Monocyte secr... (Read More) | 4 [0, 0, 0, 4] |
| 1.66 | 15 | PS11380 | SREBF-1 | SREBF-1; SREBF1; SREBP-1; SREBP1; Sterol regulatory element binding protein 1; Sterol regulatory element binding transcription factor 1; Sterol regulatory element binding protein 1s; Sterol regulatory... (Read More) | 3 [0, 0, 0, 3] |
| 1.66 | 15 | PS02760 | MIRN410 | MIRN410; hsa-mir-410; microRNA; hsa-mir-410s; microRNAs; MIR410; microRNA 410... (Read More) | 3 [0, 0, 0, 3] |
| 1.66 | 15 | PS18803 | CPE-1 | CPE-1; Flavoprotein linked monooxygenase; Xenobiotic monooxygenase; Microsomal monooxygenase; CPE1; CYP2E; CYP2E1; CYP2E1 protein; CYPIIE-1; CYPIIE1; Cytochrome P450 2E1; Cytochrome P450 family 2 subf... (Read More) | 3 [0, 0, 0, 3] |
| 1.66 | 15 | PS03717 | Breast carcinoma associated antigen DF3 | Breast carcinoma associated antigen DF3; CD227; CD227 antigen; Carcinoma associated mucin; DF3 antigen; Episialin; Epithelial membrane antigen; Epithelial mucin tandem repeat sequence; H23 antigen; H2... (Read More) | 3 [0, 0, 0, 3] |
| 1.66 | 15 | PS21265 | AIID | AIID; FOXP-3; FOXP3; Forkhead box P3; Forkhead box protein P3; IPEX; PIDX; SCURFIN; XPID; Zinc finger protein JM2; Forkhead box P3s; Forkhead box protein P3s; Zinc finger protein JM2s; immune dysregul... (Read More) | 3 [0, 0, 0, 3] |
| 1.66 | 15 | PS17035 | ADRB-3 | ADRB-3; ADRB3; ADRB3R; B3AR; BETA3AR; Beta-3 adrenergic receptor; Beta-3 adrenoceptor; Beta-3 adrenoreceptor; Beta-3 adrenergic receptors; Beta-3 adrenoceptors; Beta-3 adrenoreceptors; Beta 3 adrenerg... (Read More) | 3 [0, 0, 0, 3] |
| 1.66 | 15 | PS21135 | 65 kDa FK506-binding protein | 65 kDa FK506-binding protein; PPIase; Rotamase; FK506 binding protein 10; FK506 binding protein 10 (65 kDa); FK506-binding protein 10 precursor; FKBP10; FKBP65; Immunophilin FKBP65; PSEC0056; Peptidyl... (Read More) | 3 [0, 0, 0, 3] |
